# Deficient Phosphorylation of Stat1 in Leukocytes Identifies Neutralizing Antibodies in Multiple Sclerosis Patients Treated with Interferon-Beta

**DOI:** 10.1371/journal.pone.0088632

**Published:** 2014-02-19

**Authors:** Sonia Gavasso, Ellen Faergestad Mosleth, Tove Marøy, Katarina Jørgensen, Hanne-Linda Nakkestad, Bjørn-Tore Gjertsen, Kjell-Morten Myhr, Christian Vedeler

**Affiliations:** 1 Department of Neurology, Haukeland University Hospital, Bergen, Norway; 2 The Norwegian Multiple Sclerosis Competence Centre, Department of Neurology, Haukeland University Hospital, Bergen, Norway; 3 Department of Clinical Medicine, University of Bergen, Bergen, Norway; 4 Nofima, Ås, Norway; 5 Department of Clinical Science, Hematology Section, University of Bergen, Bergen, Norway; 6 Department of Internal Medicine, Haukeland University Hospital, Bergen, Norway; 7 The KG Jebsen Centre for MS Research, University of Bergen, Bergen, Norway; Virgen Macarena University Hospital, School of Medicine, University of Seville, Spain

## Abstract

**Background:**

Anti interferon-beta (IFN-β) neutralizing antibodies (NAb) affect efficacy of treatment of multiple sclerosis patients, but exactly when the detrimental effects of NAbs offset therapeutic efficacy is debated. Quantification of intracellular pathway-specific phosphorylation by phospho-specific flow cytometry (phosphoflow) is a promising tool for evaluation of these effects in primary immune cells from treated patients at the single-cell level.

**Method:**

Samples for phosphoflow and gene expression changes were collected before administration of IFN-β and at four, six, and eight hours thereafter. Patients were NAb negative (n = 3) or were NAb positive with low/medium (n = 1) or high (n = 2) NAb titers. Levels of phosphorylation of six Stat transcription factors (pStat) in seven cell subtypes and expression levels of 71 pathway-specific genes in whole blood were measured. The data was subjected to principal component analysis (PCA), fifty-fifty MANOVA, ANOVA, and partial least square regression (PLSR).

**Results:**

PCA of pStat levels clustered patients according to NAb class independently of time. PCA of gene expression data clustered patients according to NAb class but was affected by time and treatment. In the fifty-fifty MANOVA, NAb class was significant for both pStat levels and gene expression data. The ANOVA identified pStat1 protein in several cell subtypes as significantly affected by NAb class. The best fitting model for NAb prediction based on PLSR included pStat1 in monocytes, T cells, or lymphocytes and pStat3 in monocytes (r = 0.97). Gene expression data were slightly less predictive of NAb titers.

**Conclusion:**

Based on this proof of concept study, we hypothesize that NAb effects can be monitored by evaluation of a single biomarker, pStat1, in either monocytes or T cells by phosphoflow directly after IFN-β administration. The method will significantly reduce cost relative to labor intensive *in vitro* methods and offers a patient-specific approach to NAb evaluation.

## Introduction

Interferon-beta preparations (IFN-β) are immunogenic and development of neutralizing antibodies (NAb) to IFN-β is a significant cause of treatment failure in multiple sclerosis (MS) patients [1]. As both the appearance of NAbs and the natural course of the disease are variable and unpredictable, it has been difficult to predict when the detrimental effects of NAbs offset therapeutic efficacy. No biomarkers have been identified that correlate with IFN-β efficacy and NAb development [2]. Testing for NAbs is recommended, and several cell line based assays are used in clinical practice to guide therapeutic decisions [2], [3]. These assays detect and quantify NAbs in sera of patients, but the reported titers are in many cases not correlated with clinical outcomes. Patients who develop NAbs have no obvious adverse effects and evaluating therapy efficacy may take months to years. The effort to implement an *in vivo* gene expression assay has been hampered by the variability among patients and by the several hundred genes known to be regulated by IFN-β [4]. Only a test that incorporates the patient's own characteristics of disease and IFN-β response will reveal the effect NAbs have in individual patients.

Phospho-specific flow cytometry is an ideal platform to link the responsiveness of the IFN-β signaling pathway in primary immune cell subtypes to the effects of NAbs in individual patients [5]. We have previously developed an *ex vivo* phosphoflow assay to directly assess the impact of NAbs in primary immune cells from a patient [6], [7]. The results clearly showed that both low and high titers of NAbs detected *in vitro* significantly affected the responsiveness of immune cell subtypes to IFN-β. The activation potential of the transcription factor pStat1 was identified as a possible biomarker for NAb evaluation *ex vivo*.

In this proof of concept study we tested whether the phosphorylation and thereby activation of transcription factors of the Stats family could be measured directly after *in vivo* IFN-β administration in a patient and whether an inappropriate response to IFN-β due to NAbs could be detected and quantified immediately after treatment. We analyzed the activation of the IFN-β/Stat signaling pathway in single immune cell subtypes in whole blood from patients by phosphoflow before and at several time points after IFN-β injection. To explore whether phosphorylated Stat1 (pStat1) correlates with NAb effects *in vivo* and thus has potential as a clinical biomarker, results from the Stat phosphoflow assay were compared with analyses of IFN-β inducible gene expression in whole blood, including expression of *Mx1*, a previously proposed biomarker. This study shows, to our knowledge for the first time, that a mechanistic approach to immunogenicity evaluation is possible with phospho-specific flow cytometry. The responsiveness of the IFN-β signaling pathway after IFN-β administration showed that phosphorylated Stat1 highly correlated with NAb titers in MS patients.

## Materials and Methods

### Patients

**Figure 1:**
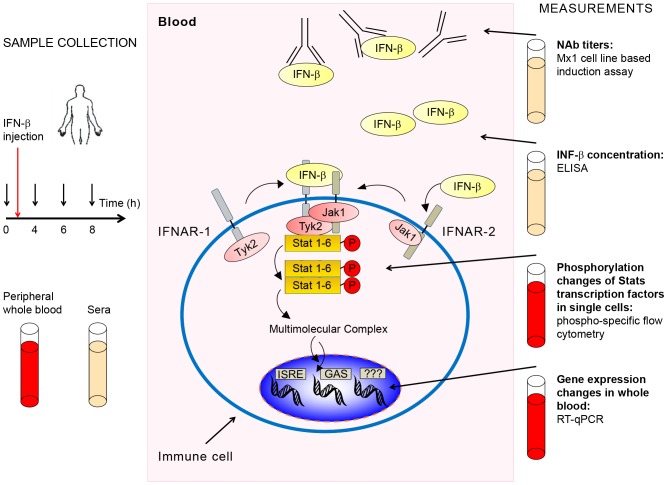
Schematic representation of workflow. The workflow diagram shows times and type of sample collection before and after IFN-β administration and sample processing and analysis. Concentrations of NAbs (against IFN-β) and IFN-β were measured in sera. mRNA used for gene expression measurements was extracted from whole blood. For signaling pathway analysis by phosphoflow, immune cell subtypes were identified and phosphorylation levels of various Stat proteins quantified in single cells. If present in sera, NAbs prevent the initiation of the IFN-β signal at the cell surface. In the absence of NAbs IFN-β binds to its cognate receptor and forms an activated receptor complex with associated kinases that phosphorylate Stat proteins at specific residues. The Jak/Stat pathway is the major signaling pathway activated by IFN-β.

Patient characteristics are shown in Table 1. The study was approved by the Regional Committee for Research Ethics (REK Vest) and written informed consent was obtained from all patients. During the study period, three NAb negative patients, two patients with high NAb titers and one patient with low/medium NAb titers being treated with INF-β-1a (Rebif) or INF-β-1b (Betaferon/Extavia) were recruited for the *in vivo* study. The NAb titer of each patient was determined by the cell line based *Mx1* induction assay: NAb-negative (<20), NAb-positive low/medium (20–300), or NAb-positive high (≥300) [8]. Patients were instructed to schedule their subsequent INF-β injection at the Department of Neurology, Haukeland University Hospital. This approach led to a minimum of 60 hours interval between the last injection and the injection that was monitored. For every patient a sample was taken before the injection (t_0_) to determine basal phosphorylation levels of Stat molecules and basal gene expression levels. Data from this time point (t_0_) was used to calculate induction levels. In preliminary experiments, samples from three NAb negative patients were evaluated for induction of Stats at time points ranging from 30 minutes to 24 hours after IFN-β injection. We determined that time points of 4 hours (t_4_), 6 hours (t_6_), and 8 hours (t_8_) after injection were optimal for measuring phosphorylation induction by phospho-specific flow cytometry and gene expression changes by RT-qPCR. Sera were collected at these time points and analyzed for IFN-β concentrations. The experiment is schematically shown in Figure 1.

**Table 1:** Patient characteristics.

Patient Identification number	Sex	Age	Disease onset	EDSS^a^	Treatment duration (years)	IFN-β preparation	NAb levels^b^
**106**	F	39	2003	0	3	IFN-β-1a s.c.	High
						Rebif	
**108**	F	40	1999	1	5	IFN-β-1b s.c.	High
						Betaferon	
**109**	M	45	1984	2	3	IFN-β-1b s.c.	Neg.
						Extavia	
**110**	F	37	1998	2,5	1	IFN-β-1b s.c.	Neg.
						Extavia	
**111**	F	44	2002	2	3	IFN-β-1a s.c.	Neg.
						Rebif	
**112**	F	49	2001	4	2	IFN-β-1a s.c.	Med.
						Rebif	

a Expanded Disability Status Scale score.

b NAb titers were measured in sera of patients by the *Mx1* induction assay.

### Single-cell quantification by flow cytometry of phosphorylated Stats signaling molecules after in vivo IFN-β administration

Samples taken at 4, 6, and 8 hours after IFN-β administration were suitable for both phosphoflow and gene expression measurements in patient blood. Whole blood was collected in sodium-heparin tubes (Terumo Venosafe – 0545H) and immediately lysed and fixed with BD Lys/Fix solution and processed according to manufacturer's instructions. Washed cells were permeabilized with ice cold 95% methanol at room temperature for 10 min and stored at −80°C. Two hours after collection of the last sample, all patient samples were washed three times and stained with indicated antibodies from BD: CD3-PE (5553333), CD4-PE-Cy7 (348809), CD8-PacificBlue (558207), CD20-PerCp-Cy5.5 (558021), CD33-PE-Cy7 (333946), and pStat1(Y701)-Alexa647 (612597) and pStat6 (Y641)-Alexa488 (612000), or pStat5 (Y694)-Alexa647 (612599) and pStat3 (Y705)-Alexa488 (557814), or pStat4 (Y693)-Alexa488 (558136) and pStat3 (S727)-Alexa647 (558099). CD94-PE (555889) was used in combination with all pStat antibodies in a separate tube. All antibodies were titrated for optimal separation and staining. Cells for every staining cocktail were obtained from an average of 200 µl whole blood. Samples were run on the BD Canto. BD Cytometry Setup and Tracking beads were used for standardization of application setup and BD compensation beads were used for electronic compensation. Compensation was evaluated by single and double stains for optimal compensation parameters. As positive controls and to assess whole blood phosphoflow *in vivo*, whole blood was re-stimulated *ex-vivo* with INF-β at a high dose for maximal phosphorylation response. In set-up experiments we determined the dose-response curve for the *ex vivo* re-stimulation response with INF-β (Avonex) in whole blood. In patients treated with INF-β-1b (Rebif) the dose-response curve ranging from 250–5000 IU/ml was analyzed.

**Figure 2:**
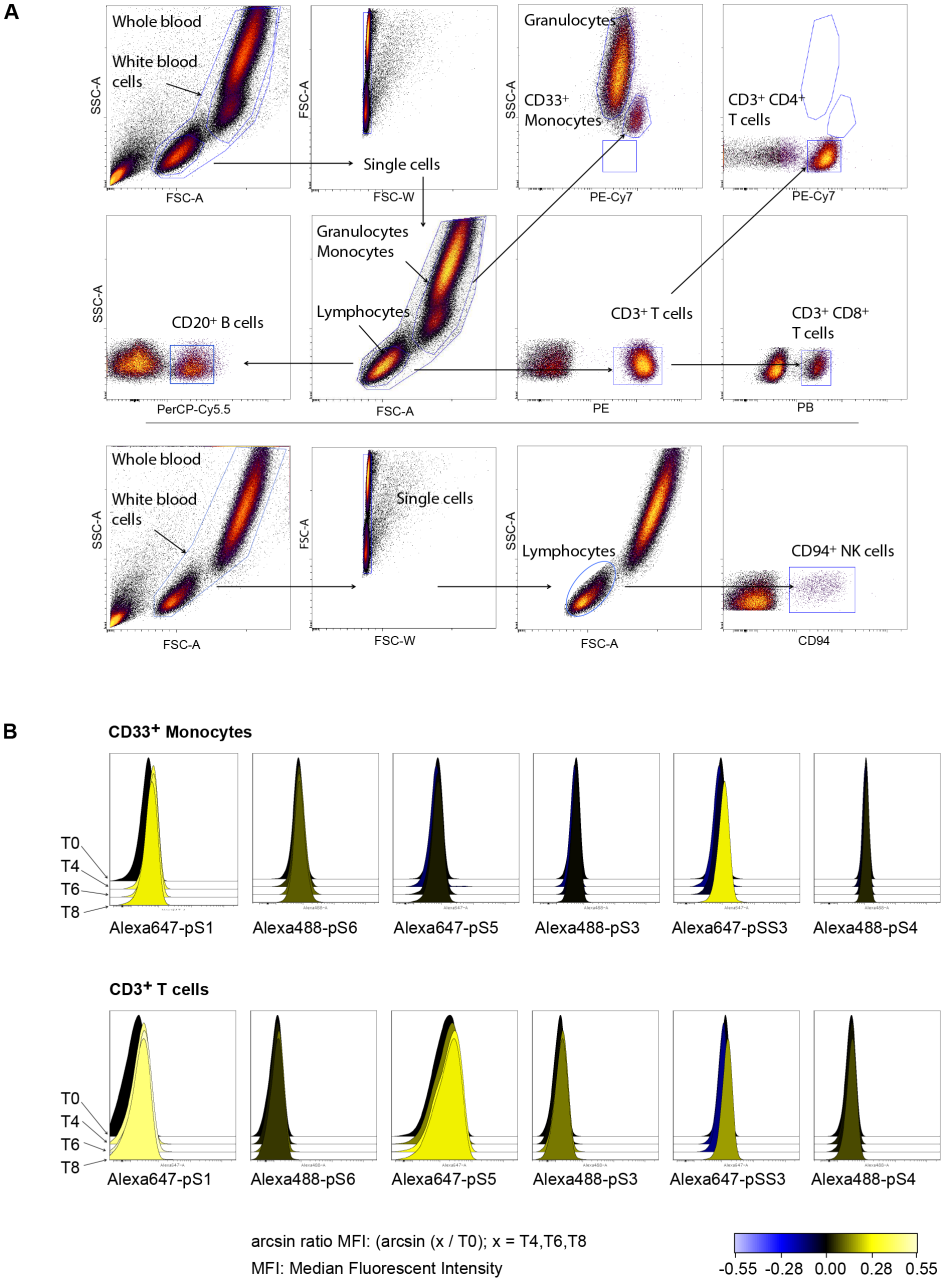
Gating hierarchy and Stats phosphorylation. (**A**) Gating hierarchy in whole blood.. An average of about 11,000 events were collected for CD33^+^ monocytes, 4500 for CD20^+^ B cells, 30,000 for CD3^+^ T cells, 20,000 for CD3^+^CD4^+^ T cells, 7500 for CD3^+^CD8^+^ T cells, and 120,000 CD33^+^ granulocytes. (**B**) Histogram overlays of fluorescent intensities of antibody conjugated fluorochromes specific for indicated phosphorylated Stat proteins are shown before and after IFN-β administration. Phosphorylation levels for Stat proteins are shown in CD33^+^ monocytes and CD3^+^ T cells. Increased intensities are represented in yellow histograms.

Eight immune cell subtypes analyzed were identified and gated as shown in Figure 2A, and an average of about 40,000 events were collected on the lymphocyte gate. Heat maps and histogram overlays were done in Cytobank software (www.cytobank.org). To display negative fluorescent intensity measurements after compensation, the inverse hyperbolic sine with a cofactor was used as recommended by Cytobank. The signal induction for IFN-β was calculated as the fold change of median fluorescent intensity (MFI) of phospho-Stat protein before injection of IFN-β in specific cell subtypes compared to MFI of samples at 4, 6, and 8 hours after administration of IFN-β in the same cell type (Figure 2B). Relative MFI calculations (MFI_(t0)_/MFI_(t4,6,8)_) were the bases for multivariate analysis.

### Gene expression changes in whole blood after in vivo IFN-β administration

The SABiosciences kit RT^2^ Profiler PCR array “Human Interferon α, β Response” (PAHS-016A) was used to measure pathway-specific gene induction after IFN-β administration *in vivo*. Blood was drawn directly into Tempus RNA Blood tubes from Invitrogen (4342792) and was stored at −80°C. The blood was processed for RNA extraction with the Spin RNA isolation kit (Applied Biosystems, 4380204). For every plate, 500 ng RNA was used for reverse transcription with SABiosciences RT^2^ First strand kit (30401). The instrument-specific Sybr green master mix from SABiosciences (330522) was used for amplification detection, and gene induction was calculated by the ΔΔCt method with SABiosciences web-based PCR array analysis tool. Every plate was checked for melting curves, DNA contamination, RNA transcription efficiency, and a positive PCR control and was passed if the values were within recommended range. The average of the five included housekeeping genes was used for normalization of data. The changes relative to levels before IFN-β administration were calculated to determine gene induction.

### IFN-β concentration in sera after in vivo IFN-β administration

The VeriKine_HS Human IFN-β Serum ELISA kit (PBL interferon source 41415-1) was used to measure levels of INF-β before IFN-β administration and at 4, 6, and 8 hours after injection. All samples were run on the same plate and concentrations were calculated according to the manufacturer's instructions.

### Data exploration, dimension reduction, and modeling

Whole data-sets were obtained for patients treated subcutaneously (s.c.) with IFN-β 1b (Extavia®/Betaferon®) or IFN-β 1a (Rebif®) of different NAb classes (negative, low/medium, and high), eight cell subtypes, six Stat phospho-proteins, and 71 genes. This set of data was used in further analysis of NAb effects. Phosphoflow values for CD20^+^ B cells from patient 106 were missing for technical reasons. Raw patient data will be made available to interested parties upon receipt of a formal request and a statement ensuring patient privacy to the Department of Neurology, Haukeland Hospital.

PCA, an unsupervised method for scaling multi-dimensional data sets, was used to explore and visualize the data. PCA is based on linear transformation of the variables to principal components (PCs) so that they can be viewed and analyzed in lower dimensional spaces with minimal possible loss of information about the variables and their correlations. PCA was performed using the algorithm NIPALS in Unscrambler software (CAMO). Missing values for patient 106 CD20^+^ B cells were imputed into the iterative estimation of the scores and the loadings allowing all data to be included. The data were mean centered and run without scaling to unit variance.

Classical multivariate analysis of variance (MANOVA) cannot be used when the number of responses exceeds the number of observations or when responses are multivariate correlated. An alternative analysis, fifty-fifty MANOVA (ffMANOVA) was developed by Langsrud et al. as a generalization of ANOVA to multivariate data. The test performs a simultaneous test of several PCs [9]. The ffMANOVA was performed in the R software (www.r-project.org). The model used included treatment, NAb class, time, and all two-way interactions between these factors as design variables and Stat protein phosphorylation inductions or gene expression changes as responses. ffMANOVA performs PCA where one design factor at the time is considered. A simultaneous test of several principal components (PCs) is performed where the components accounting for 50% of the variation is included in the model, 50% of the remaining model is used for buffer, and the rest is used for validation.

To identify significant proteins and genes, we performed both a univariate test by ANOVA where p-values were adjusted for multiple comparison by False Discovery Rate (FDR) using rotation test and by multivariate analysis using jackknife modified to bilinear data analysis as a test of the stability of the regression coefficients upon cross validation in PLS regression [10]. The ANOVA model was performed as described for the ffMANOVA analysis (NAb classes, the treatments, the one and all two-way interactions were the design parameters and the protein composition the response). To predict NAb titers, the PLS regression was performed reversed using the proteins or the genes as regressor variables and the NAb titers as response. The model was first performed using all proteins or all genes as input, and thereafter using the minimum of selected proteins or genes found significant in the ANOVA and PLS regression. The variables were not weighted.

For the prediction of NAb class based on protein data we tested several PLS regression models with subsets of Stat protein data. We were interested in the question of whether the phosphoflow data is predictive of NAb titer. As phospho-specific flow cytometry is costly we sought to identify the Stat protein or subset of Stat proteins that gave the best possible prediction of responses. PLS regression belongs to the same family of bilinear methods as PCA, but the method is supervised and based on maximizing the covariance between two sets of data or matrixes in order to find latent variables in one of the matrixes that best represents and predicts variables in the other matrix. The PLS regression was performed using mean-centering without any weighting of variables. The cross validation of these models was done on all data by leaving all measurements of samples from one patient at a time out of the analysis and using this left out sample for validation.

## Results

### Stat phosphorylation patterns distinguish patients in NAb classes

In this study, samples were collected from MS patients before IFN-β administration and at 4, 6, and 8 hours thereafter. Levels of NAbs, levels of IFN-β, phosphorylation levels of Stat proteins in immune cell subtypes, and gene expression levels in whole blood were determined (Figure 1). The eight cell subtypes analyzed were identified and gated as shown in Figure 2A. The phosphorylation induction of Stat proteins post-injection is shown in Figure 2B.

**Figure 3:**
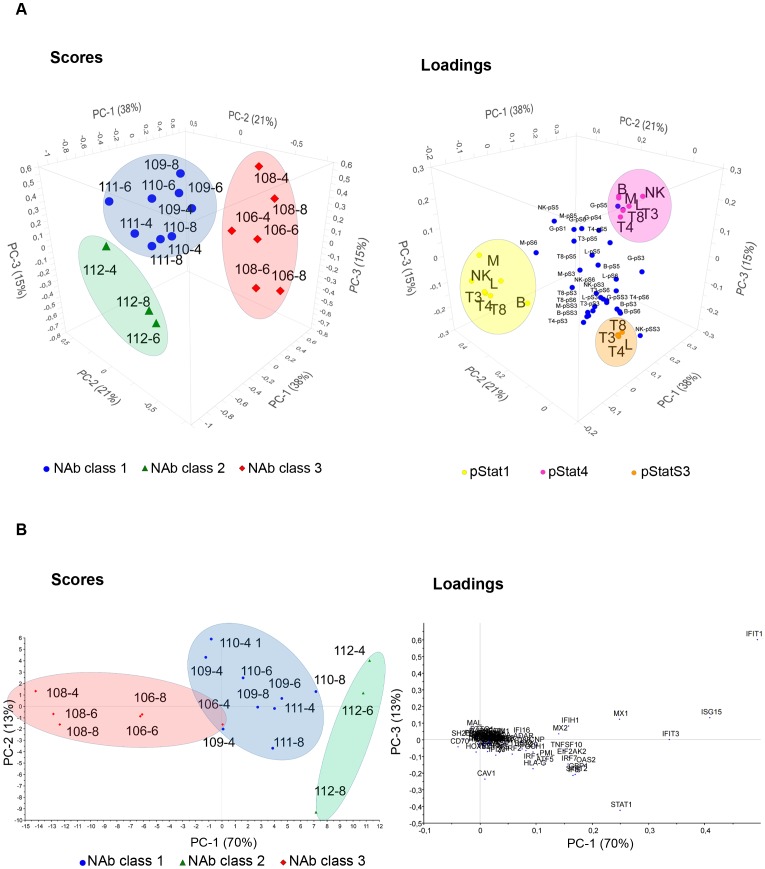
PCA of phosphorylated Stats proteins and gene expression levels. (**A**) Scores and loadings are shown for the three first PCs from the PCA of data on phosphorylation levels of Stat proteins after IFN-β administration *in vivo*. In the score plot patients cluster according to NAb classes. Patients that are NAb negative are shown with blue roundels (class 1), NAb medium patients with green triangles (class 2) and NAb high patients with red diamonds (class 3). The loading plot reveals the variables important for patient clustering. pStat1 is negatively correlated with NAb class 3, and pStat1 and pStat4 are inversely correlated. In both score plots the patients are defined by numbers according to Table 1. p-S1 through p-S6: phosphorylated Stats proteins; L: lymphocytes; T3: CD3^+^ T cells; T4: CD3^+^ CD4^+^ T cells; T8: CD3^+^ CD8^+^ T cells; B: CD20^+^ B cells; M: CD33^+^ monocytes; G: granulocytes; NK: CD94^+^ NK cells. (**B**) Scores and loadings are shown for the two first PCs from the PCA of data on gene expression changes after IFN-β administration *in vivo*. Patients cluster according to NAb class. Time of sample collection confounds clustering. The variables in the loading plot are gene names.

The unsupervised explorative multivariate analysis PCA of protein phosphorylation and gene expression revealed that the main variability in the protein phosphoflow data was related to NAb class as the NAb classes could clearly be distinguished by the three first and most important PCs in the score plot (Figure 3A and B). In this study, patients clustered according to NAb class independently of time of sample collection. The three first PCs described 74% of the total variability in the phosphoflow data. The first two PCs accounted for 59% of the total variability and distinguished patients that were NAb negative (NAb class 1) from patients that were NAb high (NAb class 3), whereas the third PC was necessary to distinguish the patient that was NAb low/medium (NAb class 2). Phosphorylated pStat1 and pStat4 were inversely correlated and, together with serine phosphorylation of Stat3 (pStatS3), were the variables that contributed the most to the clustering of patients by NAb class. No outliers were detected in the PCA.

In the scores plot, patients in NAb class 1 clustered on the upper left side of the plot (blue circle) and patients in NAb class 3 clustered on the right side of the plot (red circle). In the corresponding loading plot, pStat1 variables clustered on the left side (yellow circle) and pStat4 variables clustered on the right side (pink circle) (Figure 3A). The location and clustering of samples and variables in these plots showed that most of the cell subtypes in which phosphorylation of Stat1 was measured were negatively correlated with NAb class 3 and positively correlated with NAb class 1. In granulocytes phosphorylation of Stat1 was located near the origin (not included in the yellow circle) and therefore had no relation to NAb class. All cell subtypes for phosphorylation of Stat4 data clustered and were negatively related to NAb class 1.

**Figure 4:**
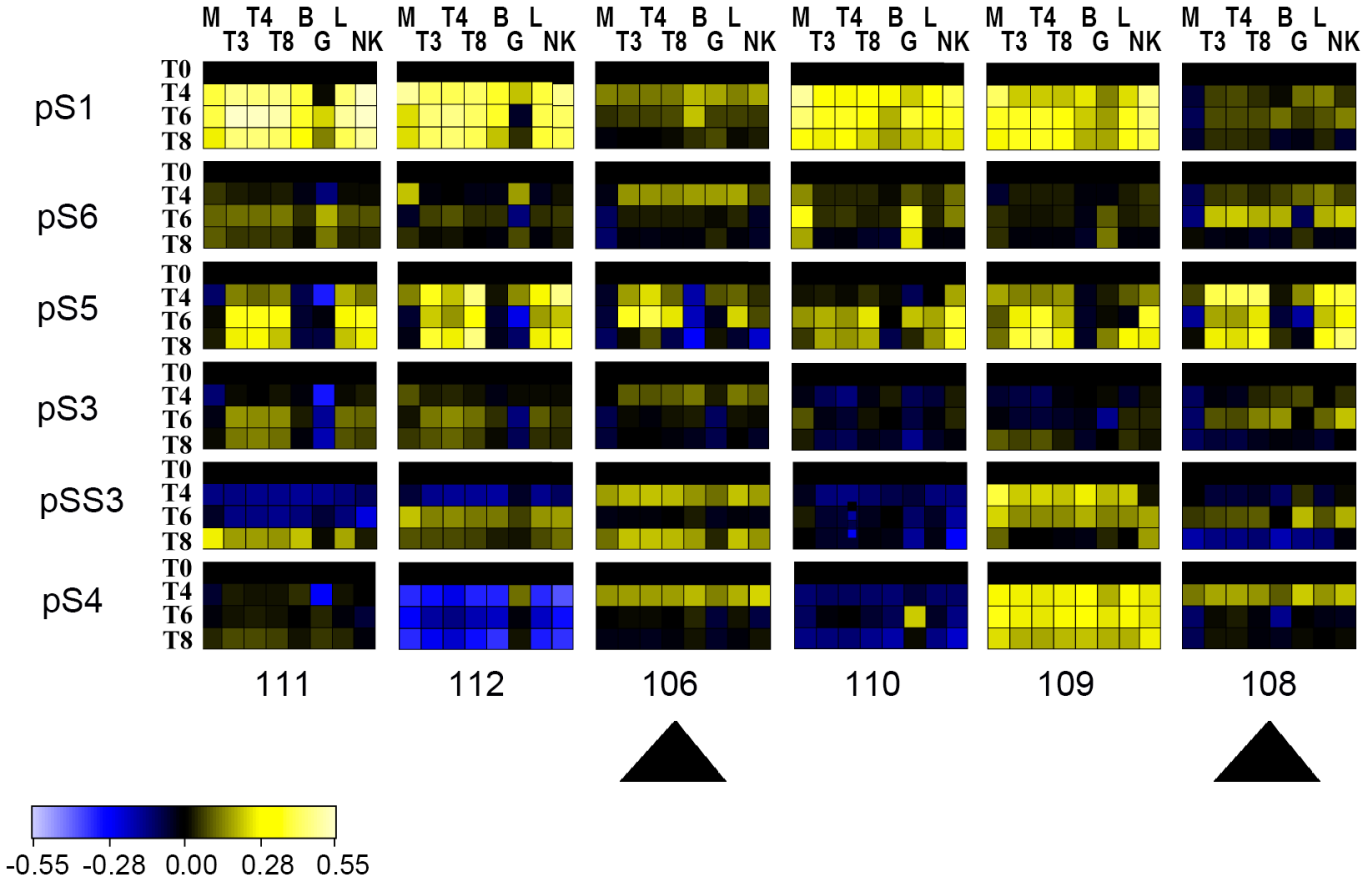
Heat maps of phosphoflow data. Heat maps indicate median fluorescent intensities (MFI) changes of phosphorylated Stat proteins in cell subtypes. The MFI before administration of IFN-β is represented in black and increases in MFI are shown in yellow and decreases in blue. Patients with high NAb titers can be distinguished based on their phosphorylation patterns (arrows). Patients are numbered according to Table 1. T0: prior to IFN-β injection; T4, T6, T8: 4, 6, and 8 hours after IFN-β administration; p-S1 through p-S6: phosphorylated Stat proteins; M: CD33^+^ monocytes; T3: CD3^+^ T cells; T4: CD3^+^CD4^+^ T cells; T8: CD3^+^CD8^+^ T cells; B: CD20^+^ B cells; L: lymphocytes; G: granulocytes; NK: CD94^+^ NK cells.

**Figure 5:**
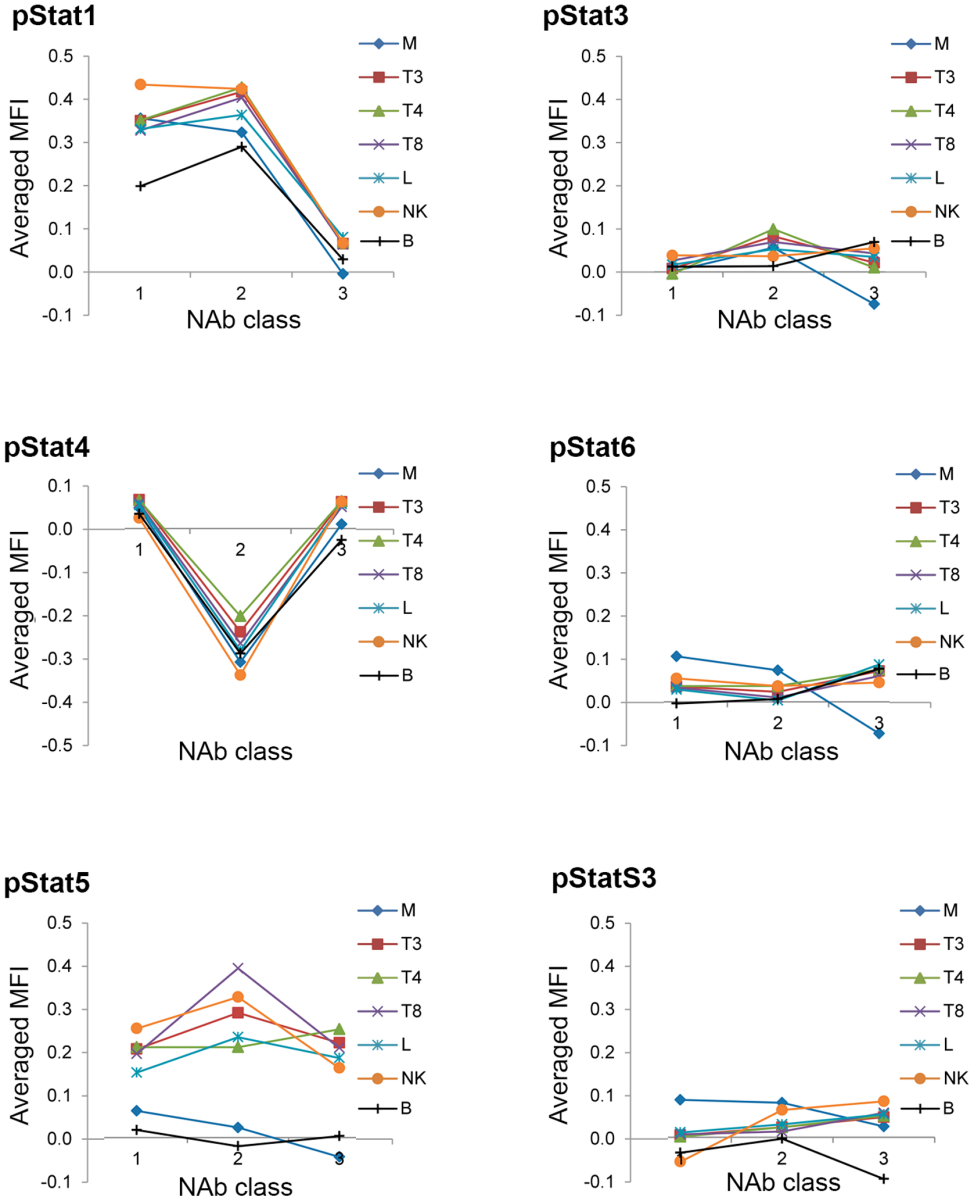
Time-averaged phosphoflow data of Stat proteins sorted by cell type and NAb class. Visualization of ffMANOVA and ANOVA results with pivot tables graphs. Phosphoflow data (stimMFI/unstimMFI) for every patient for every cell subtype was averaged for time points t_4_, t_6_, and t_8_. The main factor, time of sample collection, did not affect the phosphoflow data significantly. The importance of pStat1 and pStat4 in NAb class identification are clearly visible. M: CD33^+^ monocytes; T3: CD3^+^ T cells; T4: CD3^+^CD4^+^ T cells; T8: CD3^+^CD8^+^ T cells; B: CD20^+^ B cells; L: lymphocytes, NK: CD94^+^ NK cells. Granulocytes are not shown. MFI: median fluorescent intensity.

The relative changes in MFIs for phosphorylated Stat proteins after IFN-β injection are shown in heat maps in Figure 4. A relatively small (maximum 1.7 fold), but consistent, increase in phosphorylated Stat1 protein was observed between 4 and 8 hours after IFN-β administration in NAb-negative patients (patients 109, 110, 111). In NAb-positive patients with high titers, levels of phosphorylated Stat1 were clearly decreased compared to the levels in NAb-negative patients (106, 108) (Figure 4). Fold changes in MFIs of phosphorylated Stat proteins did not differ significantly at 4, 6, and 8 hours relative to t_0_ nor was there an interaction between the time and NAb class (Tables 2). For visualization purpose we therefore used time-averaged fold changes in phosphorylation levels for all Stat proteins and plotted these against NAb class in each cell subtype in Figure 5; these graphs illustrate the importance of pStat1 and pStat4 for the separation of NAb classes.

**Table 2:** Fifty-fifty MANOVA of Stats phosphorylation induction levels after IFN-β administration.

Factors	Df	exVarSS	nPC	nBU	exVarPC	exVarBU	p-Value
**Treatment**	1	0.05513	2	2	0.7247	0.8976	0.0452*
**Time**	1	0.02792	2	2	0.7164	0.8857	0.7192
**NAb class**	2	0.35313	2	2	0.6978	0.8983	1.19e-7***
**Trt×Time**	1	0.02801	2	2	0.7509	0.9181	0.6453
**Trt×NAb class**	1	0.07146	2	2	0.6886	0.9213	0.0510
**Time×NAb class**	2	0.07493	2	2	0.7432	0.9214	0.3821
**Residuals**	9	0.34890					

Significant codes: 0 ‘***’; 0.001 ‘**’; 0.01 ‘*’; 0.05 ‘.’.

The ffMANOVA of Stats proteins included 18 objects and 42 responses. Trt: treatment; Df: degrees of freedom; exVarSS: explained variance sum of squares; nPC: number of principal components; nBU: number of buffer components; exVarPC: explained variance in principal components; exVarBU: explained variance in buffer components.

### Gene expression patterns after in vivo IFN-β administration are heterogeneous

As expected, induction of expression of a number of genes was observed after IFN-β injection. The expression values measured are an average of whole blood mRNA. In phosphoflow, the cell subtypes can be identified and signal induction quantified without first separating the cells. To reduce the dimension and visualize gene induction we used PCA on the ΔΔCt values, which are gene expression changes relative to gene expression levels before IFN-β administration. Among the genes evaluated, *CXCL10* had a very high standard deviation and dominated the first PC in the PCA. A meaningful PCA was therefore obtained by down-weighting the data for *CXCL10* in the analysis (Figure 3B). The first two PCs explained 83% of the variance. Many genes contribute to the clustering according to NAb class, including *Mx1*.

### Markers predictive of NAb class

NAb class was significantly correlated with both Stat phosphorylation patterns and gene expression patterns after IFN-β administration *in vivo*. The significances of the PCs as analyzed by the ffMANOVA are given in Table 2 and 3 for proteins and genes, respectively. Results are presented using unscaled variables for both the Stats phosphorylation data and the gene expression changes. Two PCs were included in the model of the ffMANOVA (nPC) and two PCs were used for buffer (nBU) both for the proteins and for the genes. The main effects and the two-way interactions were included in the model. Similar to the PCA in Figure 3B, *CXCL10* was removed from the analysis. NAb class was highly significant for both the phosphoflow and gene expression data. Treatment type was also significant for both the phosphoflow data and gene expression. No significant interactions between the main factors time post injection, NAb class, or treatment type were found for phosphoflow data. The interaction between NAb class and treatment type as well as the interaction between NAb class and time post injection were significant for gene expression data. Thus, the effect of NAb class on gene expression was affected by both time of blood collection after IFN-β administration and by the treatment type as shown in the ffMANOVA.

**Table 3:** Fifty-fifty MANOVA of gene expression induction levels after IFN-β administration.

Factors	Df	exVarSS	nPC	nBU	exVarPC	exVarBU	p-Value
**Treatment**	1	0.07779	2	2	0.7067	0.8677	0.004006**
**Time**	1	0.06024	2	2	0.6414	0.8457	0.000792***
**NAb class**	2	0.51660	2	2	0.8925	0.9521	3.34e-06***
**Trt×Time**	1	0.03063	2	2	0.6110	0.8368	0.059050.
**Trt×NAb class**	1	0.07793	2	2	0.6820	0.8673	0.002589**
**Time×NAb class**	2	0.05970	2	2	0.6251	0.8365	0.007396**
**Residuals**	9	0.27776					

Significant codes: 0 ‘***’; 0.001 ‘**’; 0.01 ‘*’; 0.05 ‘.’ The ffMANOVA of the analysis of gene expression included 18 objects and 70 genes. Trt: treatment; Df: degrees of freedom; exVarSS: explained variance sum of squares; nPC: number of principal components; nBU: number of buffer components; exVarPC: explained variance in principal components; exVarBU: explained variance in buffer components.

Statistical tests on the individual variables by ANOVA using p-values adjusted for multiple comparisons by false detection rate (FDR) performed by rotation test revealed significant differences in NAb class for phosphorylation patterns in Stat1 in all cell subtypes except granulocytes (Table 4, Figure 5). In T cell subpopulations, but not in monocytes and NK cells, treatment and the interaction between treatment and NAb class were significant. B cells were not included in the ANOVA, because data on B cells was not available for one patient. The mean values for NAb class 2 (low/medium) of phosphorylation of Stat1 in these cell types were close to that of NAb class 1 (negative) and close to zero for NAb class 3 (Table 5). When the reversed model was performed using PLS regression, where the Stat proteins were the input variables and the NAb classes the response, the same Stats proteins were found to be significant using the jackknife test as in the ANOVA test: Stat1 proteins were significant in all cell subtypes except granulocytes. In addition, Stat3, Stat5, and Stat6 in monocytes and serine Stat3 in T cells were also significant by jackknife in PLS regression analysis. The predictive ability of the model including these variables was high (r = 0.89).

**Table 4:** ANOVA of pStat protein data where all proteins significant for at least one factor are presented.

Cell-type	pStat	Treatment	Time	NAb class	Trt×time	Trt×NAb class	Time×NAb class
**CD33^+^ Monocytes**	pS1	0.725	0.065	0.000*	0.971	0.142	0.639
**CD3^+^ T cells**	pS1	0.006*	0.755	0.000*	0.771	0.017*	0.554
**CD3^+^ CD4^+^ T cells**	pS1	0.008*	0.982	0.000*	0.771	0.020*	0.504
**CD3^+^ CD8^+^ T cells**	pS1	0.004*	0.742	0.000*	0.771	0.017*	0.645
**Lymphocytes**	pS1	0.008*	0.359	0.000*	0.881	0.017*	0.504
**CD94^+^ NK cells**	pS1	0.020*	0.151	0.000*	0.950	0.173	0.825

*Significant codes: <0.050.

**Table 5:** Averaged time points for pStat1 values for the three NAb classes.

pStats1 proteins in cell subtypes^a^	NAb class 1 negative	NAb class 2 low	NAb class 3 high
**CD33^+^ Monocytes**	0.36	0.32	0.00
**CD3^+^ T cells**	0.35	0.42	0.07
**CD3^+^ CD4^+^ T cells**	0.35	0.43	0.07
**CD3^+^ CD8^+^ T cells**	0.33	0.40	0.07
**Lymphocytes**	0.33	0.36	0.08
**CD94^+^ NK cells**	0.43	0.42	0.07

a Phosphorylation levels of pStats1 are time averaged.

A statistical test on individual genes by ANOVA using FDR identified many genes that were significantly affected by NAbs in whole blood after IFN-β injection. These genes are shown in Table 6. For the genes *ISG15*, *MX1*, and *MX2* there was a significant interaction between NAb class and treatment, and for the genes *CNP* and *IFI16* there was a significant interaction between treatment and time. The mean values of significant ANOVA results for each NAb class are presented in Table 7.

**Table 6:** ANOVA of gene data where all genes significant for at least one factor are presented.

Gene name	Treatment	Time	NAb class	Trt×Time	Trt×NAb class	×NAb class
ADAR	**0.032** *	0.843	**0.005** *	0.337	0.298	0.521
ARL5B	0.261	0.480	**0.005** *	0.096	0.481	0.731
ATF5	0.377	0.059	**0.003** *	0.115	0.759	0.273
BAG3	0.779	0.964	**0.048** *	0.182	0.757	0.752
BST2	0.547	0.225	**0.041** *	0.119	0.641	0.521
CASP1	**0.036** *	0.055	**0.015** *	0.096	0.356	0.752
CDKN1B	**0.044** *	0.820	0.793	0.461	0.641	0.921
CNP	**0.002** *	0.731	**0.000** *	**0.047** *	0.834	0.273
EIF2AK2	**0.031** *	0.625	**0.005** *	0.096	0.248	0.487
GBP1	**0.001** *	**0.004** *	**0.000** *	0.461	0.641	0.273
GCH1	**0.032** *	0.292	**0.003** *	0.385	0.622	0.816
HLA_DOA	**0.048** *	0.335	0.336	0.156	0.641	0.723
HLA_DQA1	0.558	0.501	**0.041** *	0.273	0.615	0.521
HLA_F	**0.008** *	0.225	**0.036** *	0.260	0.896	1.000
HLA_G	0.405	0.466	**0.048** *	0.973	0.645	0.902
IFI16	**0.005** *	0.181	**0.000** *	**0.047** *	0.298	0.816
IFI27	**0.024** *	0.055	0.412	0.973	0.330	0.816
IFI6	**0.044** *	**0.004** *	**0.000** *	0.182	0.080	0.273
IFIH1	0.065	0.507	**0.000** *	0.337	0.084	0.731
IFIT1	0.410	0.068	**0.000** *	0.808	0.307	0.273
IFIT3	**0.048** *	0.650	**0.000** *	0.260	0.084	0.816
IFITM2	**0.004** *	**0.035** *	**0.002** *	0.461	0.946	0.816
IFNAR2	**0.009** *	0.869	0.913	0.973	0.641	0.746
IRF1	**0.024** *	0.501	**0.015** *	0.461	0.977	0.731
IRF2	**0.041** *	0.549	**0.019** *	0.280	0.641	0.816
IRF7	**0.010** *	0.702	**0.015** *	0.125	0.387	0.411
IRF9	**0.005** *	0.564	**0.009** *	0.521	0.697	0.521
ISG15	0.581	0.181	**0.000** *	0.096	**0.006** *	0.430
ISG20	0.034	0.160	**0.004** *	0.096	0.918	0.723
MAL	0.009	0.977	0.784	0.193	0.641	0.273
MX1	0.276	0.323	**0.000** *	0.096	**0.006** *	0.521
MX2	0.622	0.225	**0.000** *	0.096	**0.006** *	0.521
MYD88	**0.031** *	0.731	**0.015** *	0.115	0.834	0.815
NPEPPS	**0.024** *	0.869	0.729	0.096	0.298	0.997
OAS2	0.051	0.486	**0.004** *	0.260	0.558	0.487
PML	**0.034** *	0.708	**0.014** *	0.521	0.760	0.816
PSME2	0.268	**0.004** *	**0.048** *	0.157	0.641	0.816

*Significant codes: <0.050.

**Table 7:** Averaged time points for gene expression values for the three NAb classes.

Genes^a^	NAb class 1 negative	NAb class 2 low	NAb class 3 high
**ADAR**	2.17	2.19	1.21
**ARL5B**	1.19	1.73	1.04
**ATF5**	2.22	3.66	1.26
**BAG3**	0.97	1.63	1.00
**CASP1**	1.49	1.59	1.04
**CD70**	1.10	2.21	2.11
**CNP**	2.68	2.88	1.43
**EIF2AK2**	2.74	4.70	1.76
**GBP1**	3.53	4.96	1.47
**GBP2**	1.70	1.73	1.14
**GCH1**	2.57	2.79	1.24
**HLA.F**	1.12	1.13	0.95
**HLA.G**	1.56	3.52	0.86
**IFI16**	2.05	2.48	1.21
**IFI6**	3.62	5.30	2.14
**IFIH1**	3.57	3.90	1.37
**IFIT1**	8.51	11.98	2.49
**IFIT3**	5.98	8.83	2.43
**IFITM2**	1.36	1.35	0.97
**IRF1**	1.98	3.27	1.31
**IRF2**	1.55	2.44	1.14
**IRF7**	3.49	4.39	2.09
**IRF9**	1.61	1.92	1.18
**ISG15**	8.19	9.52	2.80
**ISG20**	2.18	2.64	1.27
**MX1**	5.26	6.30	2.38
**MX2**	3.35	3.51	1.55
**MYD88**	1.82	2.27	1.23
**OAS2**	3.18	5.66	1.84
**PML**	2.52	3.54	1.29
**PSME2**	1.28	1.65	1.21
**SH2D1A**	0.90	0.68	1.24
**STAT1**	3.73	7.49	1.96
**STAT2**	3.07	5.03	1.65
**TAP1**	1.42	1.70	0.99
**TNFSF10**	3.53	4.01	1.38

a Gene names.

Trt: treatment type.

### Stat1 phosphorylation predicts NAb titers after in vivo IFN-β administration

**Figure 6:**
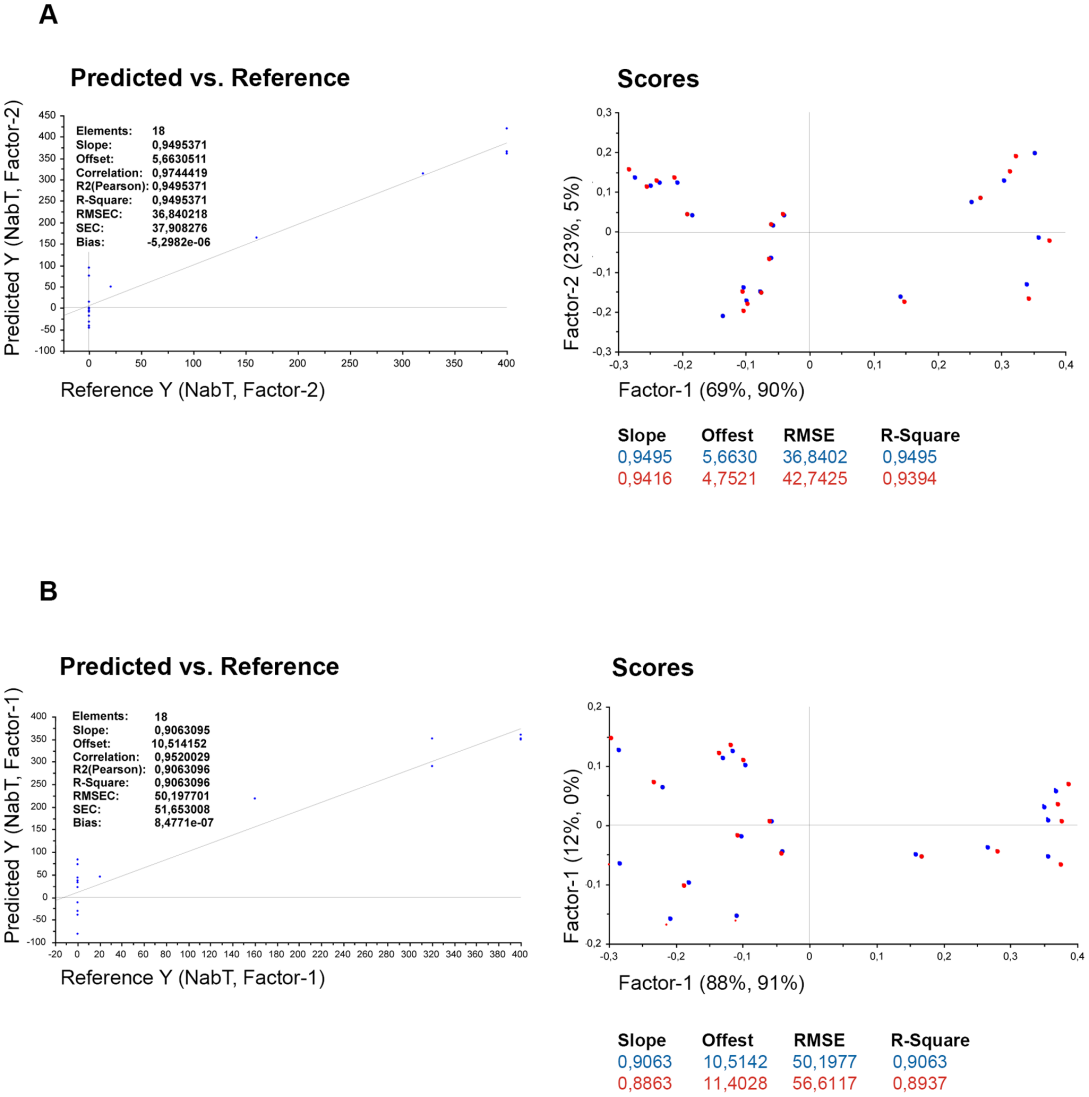
PLS model of pStat1 in CD33^+^ monocytes and lymphocytes and pStatS3 lymphocytes and PLS model of pStat1 in CD33^+^ monocytes and CD3^+^ T cells. (**A**) PLS model for best prediction of NAb titers (NabT) is shown in the predicted versus reference plot. The predictor variables were pStat1 in CD33^+^ monocytes and lymphocytes and pStatS3 in lymphocytes; the response variable was NabT. In the scores plot, 95% of the variability in NabT is explained by 92% of the variability in the predictor variables using the first two factors. The blue circles represent samples for the calibration setup model, and the red circles represent the validation samples obtained by full cross validation. (**B**) PLS of model of interest where pStat1 in CD33^+^ monocytes and CD3^+^ T cells are used as predictors and NabT as response variables. RMSEC: root mean square error of calibration (blue) and of validation (red), respectively; SEC: standard error of calibration and is similar to RMSEC, except it is corrected for bias which is the mean value over all points that either lie systematically above (or below) the regression line. A value close to zero indicates a random distribution of points about the regression line.

The possible application of Stat protein phosphorylation data to predict NAb class was evaluated using a prediction model based on PLSR. Time of sampling was not significant for the Stat phosphorylation data as shown in Table 2. This indicates that all three time points were equally well suited for the prediction of NAb class. Time of sampling was significant for gene expression data (Table 3). The best fitting model for the phosphoflow data included the variables pStat1 in monocytes and lymphocytes and pStat3 (at serine) in lymphocytes. The predictability of NAb titers was r = 0.97 (Figure 6A). Note that one patient at a time was excluded when calibrating the model and used for validation, which means that the NAb class on one patient is predicted based on the model obtained on the remaining patients. The phosphorylation induction of only Stat1 in two cell subtypes, namely CD33^+^ monocytes and CD3^+^ T cells or lymphocytes, was predictive of NAb titers with an accuracy of r = 0.95 (Figure 6B). pStat1 in these cell subtypes was negatively correlated with NAb titers. The same PLS model for all proteins and cell subtypes had a similar correlation coefficient (r = 0.95).

**Figure 7:**
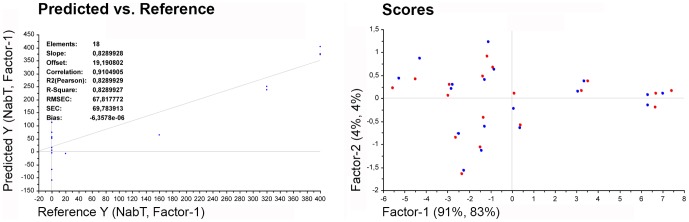
PLS model for genes *Mx1*, *TNFSF10*, *IFIT3*, and *IFIH1*. PLS model for best prediction of NAb titers (NabT) is shown in the predicted versus reference plot. The predictor variables were *Mx1*, *TNFSF10*, I*FIH1*, and *IFIT3* expression, the response variable was NabT. In the scores plot, 87% of the variability in NabT is explained by 96% of the variability in the predictor variables using the first factor. The blue circles represent samples for the calibration setup model and the red circles represent the validation samples obtained by full cross validation. RMSEC: root mean square error of calibration (blue) and of validation (red); SEC: standard error of calibration.

PLS models of gene expression changes were less predictive of NAb titers than models based on Stat phosphorylation. When all genes were included in the model none of the genes were found to be significant by the jackknife test and the predictive value of this model (r = 0.73) was lower than that based on Stat phosphorylation. When only significant genes from the ANOVA test were included in the model the correlation coefficient was r = 0.89. The best fitting model was obtained with four genes, *Mx1*, *TNFSF10*, *IFIT3*, and *IFIH1* (r = 0.91; Figure 7).

### Phospho-protein induction patterns clearly separate NAb classes in subcutaneously IFN-β-1a treated patients

**Figure 8:**
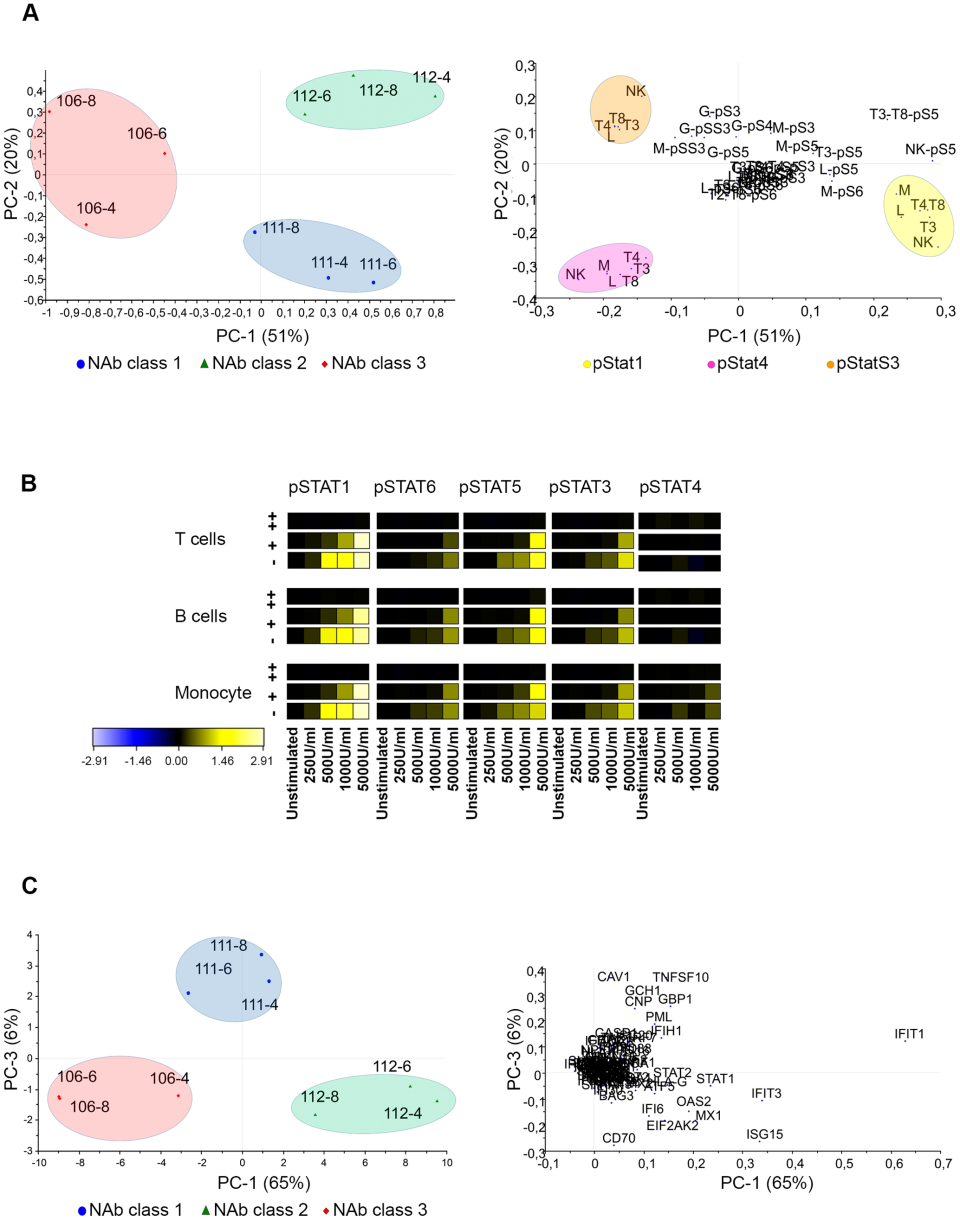
IFN-β-1a (Rebif) treatment analyzed separately. (**A**) PCA of pStat proteins in patients treated with Rebif. NAb classes form distinct clusters for all measurements. The most important variables are highlighted in the loading plot. (**B**) Heat map dose-response curves of *ex vivo* re-stimulation with IFN-β in whole blood of the same patients. Patients are in different NAb classes: NAb negative (−), NAb medium (+), and NAb high (++). Unstimulated samples were used as reference samples and are shown in black. Increases in phosphorylation are shown in yellow. (**C**) PCA of genes in patients treated with IFN-β-1a (Rebif). For clustering according to NAb classes based on gene expression changes PC1 vs. PC2 were not useful. However, PC1 vs. PC3 did cluster patients according to NAb class. The first number in the identification of patients in the score plot is the patient number according to Table 1, and the second is the time of sample collection after IFN-β injection in hours. M: CD33^+^ monocytes; T3: CD3^+^ T cells; T4: CD3^+^CD4^+^ T cells; T8: CD3^+^CD8^+^ T cells; Cd20^+^ B cells; L: lymphocytes; G: granulocytes; NK: CD94^+^ NK cells; pS1 through pS6: pStat1 through pStat6.

The ffMANOVA test showed a significant difference between patients treated with s.c. IFN-β-1a or s.c. IFN-β-1b, based on the Stat phosphorylation data (Table 2,4). This treatment effect may confound some of our PCA models. We therefore analyzed the data from patients treated with the IFN-β preparation Rebif separately. For this treatment, data on patients in all three NAb classes were available. The PCA of the phospho-protein data showed three distinct clusters for the patients with different NAb classes based on the two first PCs and explained 71% of the variation within the data (Figure 8A). pStat1, pStat4, and pStat3 (serine) were the most important variables in clustering of patients; this was similar to the PCA of Stat data when all patient data was included (Figure 3A).

To evaluate effects of NAb on the responsiveness of the IFN-β/Stats signaling pathway, whole blood from the same s.c. IFN-β-1a treated patients was re-stimulated *ex vivo* with a serial dilution of IFN-β. The dose response curves are shown in heat maps in Figure 8B. NAb titers affected the sensitivity of the pathway. The PCA for the gene induction data of the same patients is shown in Figure 8C. With the first two PCs no clustering of patients according to NAb class was observed. PC1 and PC3, however, separated NAb classes and explained 71% of the variation in the data.

### Serum values

**Figure 9:**
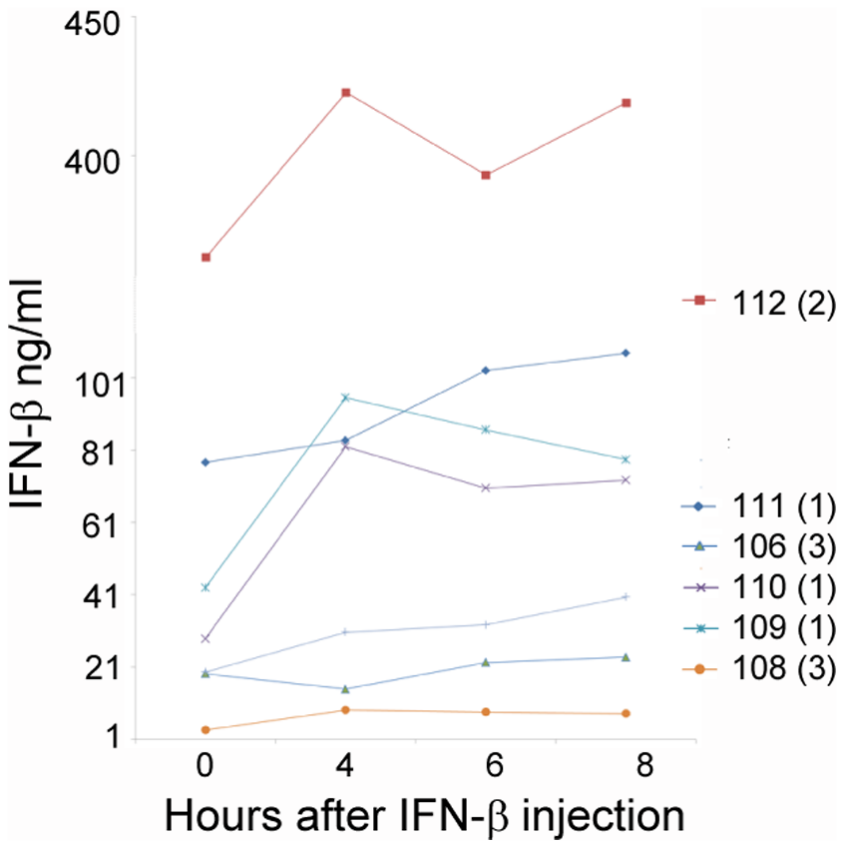
IFN-β levels in sera before and after IFN-β administration. Levels of IFN-β in sera of patient numbered according to Table 1. Numbers in parenthesis are NAb classes (1: NAb negative; 2: NAb medium; 3: NAb high).

Serum levels of IFN-β increased after treatment in NAb negative patients (Figure 9). In sera of patients, levels of IFN-β before injection tended to be higher than in healthy controls, which are either undetectable or very low in the range of 2–6 pg/ml (PBL interferon source). In patients with high NAb titers the IFN-β concentration measured in sera were very similar before and after injection.

**Figure 10:**
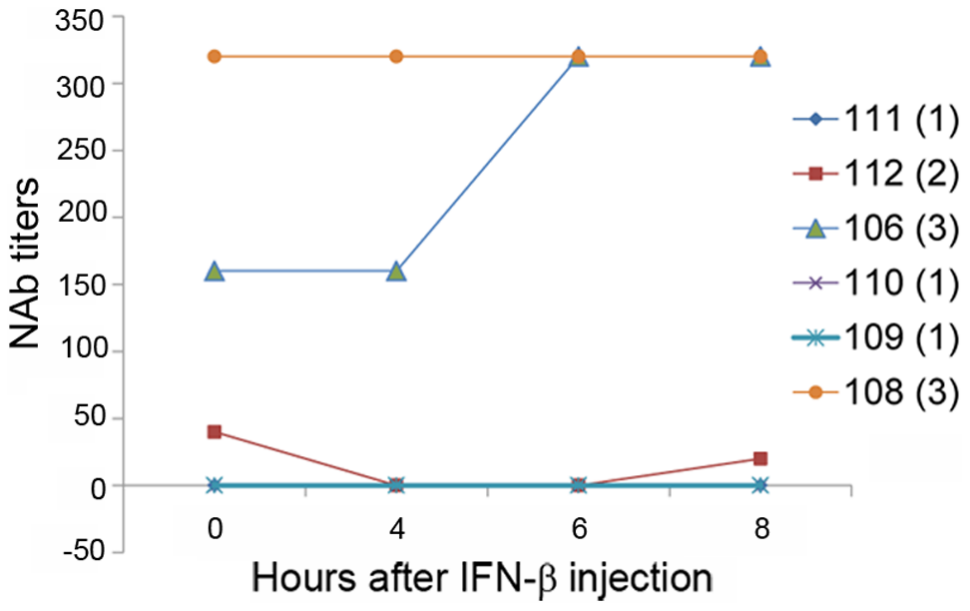
NAb titer levels in sera of patients after IFN-β administration. NAb titer levels measured with the *Mx1* induction assay. Patient numbers are based on those in Table 1. Numbers in parenthesis are NAb classes (1: NAb negative; 2: NAb medium; 3: NAb high).

Serum NAb titers measured by the *Mx1* induction assay were dependent on time of sample collection (Figure 10). Only one patient (108) had high and consistent NAb titers at every time point before IFN-β administration and thereafter. NAb negative patients remained NAb negative at every time point. A patient (112) with low/medium NAb values fluctuated between negative and positive values depending on time of sample collection.

## Discussion

INF-β is one of the first-line treatment options for relapsing-remitting MS. During therapy, up to 40% of patients develop NAbs that may affect treatment efficacy [11], [12]. In 2010 an international consensus panel of MS experts concluded that persistently high titers of NAbs are likely to be associated with treatment failure [1]. However, the clinical impact of NAb titers is controversial partly because of the lack of relevant tests that take a patient's constitution and disease variability into consideration. Some patients are at risk of being treated for months to years with limited or lost efficacy. In this proof of concept study we found that interrogation of the IFN-β signaling pathway in single cells of leukocyte subsets revealed cellular responses early after IFN-β administration and demonstrated that the pattern of Stat activation is inhibited and/or modulated in NAb positive patients and that the determination of Stat1 phosphorylation alone is a promising *in vivo* IFN-β activity biomarker.

The phosphorylation level of transcription factor Stat1 (pStat1) correlated with NAb titers. The best model for prediction of NAb titers after *in vivo* IFN-β injection was based on pStat1 in monocytes and in CD3^+^ T cells. Similarly, *ex vivo* re-stimulation of peripheral blood mononuclear cells (PBMCs) from patients with IFN-β showed that pStat1 levels in monocytes and CD3^+^ T cells contributed significantly to the clustering of patients according to NAb classes [7]. Thus, *ex vivo* IFN-β re-stimulation data and data obtained after *in vivo* IFN-β injection both indicate that pStat1 is a promising biomarker for the accurate assessment of NAb effects in individual patients.

Quantification of intracellular pathway-specific phosphorylation levels better predicted NAb effects than gene expression changes as levels of phosphorylated Stat proteins were more consistent and less heterogeneous than changes in gene expression. Gene expression was influenced by time of sample collection and by treatment type. We previously showed that in PBMCs from healthy people and in PBMCs from MS patients, the INF-β/Stat signal transduction pathway is functional when the cells are re-stimulated *ex vivo* with IFN-β in media without patient sera [7]. We consistently observed cell-type specific activation of Stat proteins in healthy individuals and untreated MS patients, and signaling patterns were inhibited and/or modulated in the presence of patient sera containing NAbs. Based on this more homogeneous *ex vivo* signal activation pattern in PBMCs it may not be surprising that phosphoflow data was more robust in predicting NAb effects than were gene expression changes after *in vivo* IFN-β injection.

Gene induction was measured for genes known to be involved in the response to IFN-β in humans. Twenty-one of the 36 genes that were significant in the ANOVA were also significant in PLSR, and ten of these genes had a high correlation to NAb titers: *ADAR*, *CNP*, *IFIT1*, *IFIT3*, *IFI16*, *ISG15*, *Mx1*, *Mx2*, *MYD88*, and *TNFSF10*. All of these showed marked down regulation in patients with high NAb levels compared to NAb negative patients. These results support the findings by Hesse et al. that in patients who lack *Mx1* expression in whole blood after INF-β administration also lack expression of other IFN-β-inducible genes [13]. The differences between NAb negative patients and the patient with low/medium NAb titers were heterogeneous and possibly affected by the large amount of endogenous IFN-β in the patient with low/medium NAb titer. The multi-level approach in this study elucidates the complexity of therapy response in patients but also shows that specific measurements within the cascade that affects signaling responses due to therapeutic agents may be used to interpret results and guide decisions regarding therapy.

Measuring phosphorylation levels of pathway-specific transcription factors may be more reliable than monitoring gene expression because the measured signals are directly linked to the activation of the IFN-β receptor at the cell surface. Although all IFN-β signals are initiated with receptor dimerization, mechanisms of associated kinase activation and phosphorylation of transcription factors, integration of this signals are complex and dependent on many factors in time and space such as activation state of cells and cross-talk between different signal inputs. The time frame of sample collection (hours) may influence Stat protein levels per se and the differences in NAb class may also be explained by translational changes. Unphosphorylated protein measurements that could discern between direct phosphorylation and translation changes were not included in this study.

IFN-β can induce hundreds of genes and the *in vivo* response to IFN-β injection assessed in blood immune cells is heterogeneous both in gene specificity and sensitivity. Nevertheless, some genes were specifically regulated in response to INF-β. The *myxovirus resistant protein 1* (*Mx1*) gene has been proposed as an *in vivo* biomarker for the responsiveness of immune cells to INF-β [13]–[15]. This *in vivo* biomarker appears to correlate with IFN-β responsiveness in healthy individuals and in many MS patients. The *Mx1* response in patients is heterogeneous, however, and arbitrary cutoffs are used to evaluate NAb effects *in vivo*.

Interestingly, the IFN-β concentrations in sera varied significantly between patients prior to the IFN-β injection. In some patients the INF-β concentrations were very low, similar to those observed in healthy people, and in other patients INF-β was clearly present in sera before the injection. The data showed an increase in IFN-β in sera of NAb-negative patients after INF-β injection, but not in NAb-positive patients. The different medication regimes, as indicated by the significant treatment effect, may explain the accumulation of IFN-β in sera of some patients but other factors may influence IFN-β concentration as well. Some of the components affecting the *in vivo* measurements in patients may be the endogenous activation of the IFN pathway by viral infection, INF-β receptor concentrations at the cell surface, and the disease itself. High amounts of IFN-β were measured in sera of patient 112. The levels of 400 ng/ml are in the range of viral infection (personal communication from ELISA manufacturer). This patient had low/medium NAb titers and how high endogenous IFN-β levels affect treatment is not known. We cannot rule out that this patient clustered separately from patients with high NAb levels because of this effect. Our data highlights the intricacy of the *in vivo* response in patients and indicates that the *in vivo* assay may also be valuable in detecting patients with an abnormal response to IFN-β for other reasons than NAb.

In the *in vitro* Mx1 NAb assay, different NAb titers were dependent on time after injection. As previously shown in many studies, the time of sampling can confound measurements and must be carefully chosen both in experimental settings and also for routine analysis. Based on our analysis, a universal biomarker for the overall INF-β response in MS patients remains elusive. However, our data indicate that a single biomarker, pStat1, may be appropriate for the evaluation of adverse NAb effects. We showed that even very low/medium NAb titers inhibited the responsiveness of PBMCs and immune cells in whole blood to IFN-β re-stimulation *ex vivo*. Thus, levels of phosphorylated Stat1 correlated with NAb titers both, after IFN-β re-stimulation *ex vivo* and after IFN-β administration *in vivo*.

In conclusion, measurements of pathway-specific activation levels of signaling molecules after *in vivo* IFN-β injection are possible, and phosphorylation patterns of Stat proteins are clearly affected by NAbs. Patient variability in Stat protein phosphorylation was less than variability in gene expression data and was, therefore, more reliable for NAb effect evaluation. We hypothesize that NAb effects are detrimental to IFN-β therapy and that these effects can be evaluated with a single biomarker pStat1. However, the actual individual response to INF-β may require measuring a panel of different signal transduction proteins. Further, our findings indicate that multivariate analysis using tools such as PCA and PLSR can be used to discern signals from noise and to identify variables crucial to the system studied.

## References

[1] Polman CH, Bertolotto A, Deisenhammer F, Giovannoni G, Hartung HP, et al (2010) Recommendations for clinical use of data on neutralising antibodies to interferon-beta therapy in multiple sclerosis. Lancet Neurol 9: 740–750. 20610349 10.1016/S1474-4422(10)70103-4

[2] Killestein J, Polman CH (2011) Determinants of interferon beta efficacy in patients with multiple sclerosis. Nat Rev Neurol 7: 221–228. 21364522 10.1038/nrneurol.2011.22

[3] Hartung HP, Kieseier B, Goodin DS, Arnason BG, Comi G, et al (2012) Variability in detection and quantification of interferon beta-1b-induced neutralizing antibodies. J Neuroinflammation 9: 129. 22703536 10.1186/1742-2094-9-129 PMC3403940

[4] Goertsches RH, Zettl UK, Hecker M (2011) Sieving treatment biomarkers from blood gene-expression profiles: a pharmacogenomic update on two types of multiple sclerosis therapy. Pharmacogenomics 12: 423–432. 21449680 10.2217/pgs.10.190

[5] Gavasso S, Myhr KM, Vedeler C (2006) Multiplexed phosphoprotein analysis in immune cells. Acta Neurol Scand Suppl 183: 58–60. 10.1111/j.1600-0404.2006.00618.x 16637932

[6] Gavasso S (2009) Flow cytometry and cell activation. Methods Mol Biol 514: 35–46. 19048212 10.1007/978-1-60327-527-9_4

[7] Gavasso S, Gjertsen B, Anderssen E, Myhr K, Vedeler C (2012) Immunogenic effects of recombinant interferon-beta therapy disrupt the JAK/STAT pathway in primary immune cells from patients with multiple sclerosis. Mult Scler 18: 1116–1124. 22287540 10.1177/1352458511434066

[8] Aarskog NK, Maroy T, Myhr KM, Vedeler CA (2009) Antibodies against interferon-beta in multiple sclerosis. J Neuroimmunol 212: 148–150. 19467718 10.1016/j.jneuroim.2009.04.012

[9] Langsrud O (2005) Rotation tests. Statistics and Computing 15: 53–60.

[10] Martens H, Martens M (2000) Modified Jack-knife estimation of parameter uncertainty in bilinear modelling by partial least squares regression (PLSR). Food Quality and Preference 11: 5–16.

[11] Dubois BD, Keenan E, Porter BE, Kapoor R, Rudge P, et al (2003) Interferon beta in multiple sclerosis: experience in a British specialist multiple sclerosis centre. J Neurol Neurosurg Psychiatry 74: 946–949. 12810786 10.1136/jnnp.74.7.946 PMC1738542

[12] Deisenhammer F (2009) Neutralizing antibodies to interferon-beta and other immunological treatments for multiple sclerosis: prevalence and impact on outcomes. CNS Drugs 23: 379–396. 19453200 10.2165/00023210-200923050-00003

[13] Hesse D, Sellebjerg F, Sorensen PS (2009) Absence of MxA induction by interferon beta in patients with MS reflects complete loss of bioactivity. Neurology 73: 372–377. 19652141 10.1212/WNL.0b013e3181b04c98

[14] Sominanda A, Hillert J, Fogdell-Hahn A (2008) In vivo bioactivity of interferon-beta in multiple sclerosis patients with neutralising antibodies is titre-dependent. J Neurol Neurosurg Psychiatry 79: 57–62. 17911184 10.1136/jnnp.2007.122549

[15] Pachner AR, Warth JD, Pace A, Goelz S (2009) Effect of neutralizing antibodies on biomarker responses to interferon beta: the INSIGHT study. Neurology 73: 1493–1500. 19884577 10.1212/WNL.0b013e3181bf98db

